# A protocol combining multiphoton microscopy and propidium iodide for deep 3D root meristem imaging in rice: application for the screening and identification of tissue-specific enhancer trap lines

**DOI:** 10.1186/s13007-018-0364-x

**Published:** 2018-10-29

**Authors:** Charlotte Bureau, Nadège Lanau, Mathieu Ingouff, Boukhaddaoui Hassan, Anne-Cécile Meunier, Fanchon Divol, Rosie Sevilla, Delphine Mieulet, Anne Dievart, Christophe Périn

**Affiliations:** 1 CIRAD, UMR-AGAP, Université de Montpellier, Avenue Agropolis, 34398 Montpellier Cedex 5, France; 2 UMR DIADE, Université de Montpellier, 911 Avenue Agropolis, 34394 Montpellier Cedex 5, France; 3 INSERM U1051, Institut des Neurosciences de Montpellier, 34095 Montpellier, France; 4 UMR Biochimie et Physiologie Moléculaire des Plantes, INRA, Campus INRA/SupAgro, 2 Place Viala, 34060 Montpellier Cedex 2, France

**Keywords:** Rice, Root meristem, Multiphoton microscope, GFP, YFP, CFP, Propidium iodide

## Abstract

**Background:**

The clear visualization of 3D organization at the cellular level in plant tissues is needed to fully understand plant development processes. Imaging tools allow the visualization of the main fluorophores and in vivo growth monitoring. Confocal microscopy coupled with the use of propidium iodide (PI) counter-staining is one of the most popular tools used to characterize the structure of root meristems in *A. thaliana*. However, such an approach is relatively ineffective in species with more complex and thicker root systems.

**Results:**

We adapted a PI counter-staining protocol to visualize the internal 3D architecture of rice root meristems using multiphoton microscopy. This protocol is simple and compatible with the main fluorophores (CFP, GFP and mCherry). The efficiency and applicability of this protocol were demonstrated by screening a population of 57 enhancer trap lines. We successfully characterized GFP expression in all of the lines and identified 5 lines with tissue-specific expression.

**Conclusions:**

All of these resources are now available for the rice community and represent critical tools for future studies of root development.

**Electronic supplementary material:**

The online version of this article (10.1186/s13007-018-0364-x) contains supplementary material, which is available to authorized users.

## Background

3D imaging technologies have become critical tools for analyzing developmental processes in plants and animals over the past 10 years. mRNA and protein mapping at a cellular or even subcellular scale is now a routine process in numerous laboratories since the discovery and demonstration of the usefulness of GFP as a biomarker [1, 2]. Among the range of techniques available, propidium iodide (PI) staining combined with the visualization of *Arabidopsis thaliana* living root tissues via confocal microscopy is one of the most popular techniques (see, for instance, [3]). The *A. thaliana* root tips are simply soaked in a PI solution, rinsed and then imaged directly with a confocal microscope. PI, which is a vital stain, has made it possible to carry out in vivo observations, such as cell ablation [3], time-lapse cell divisions inside the root meristem [4, 5] or a mosaic analysis of SCR transcription factor function [5]. Despite its simplicity and popularity in many labs, the combination of confocal microscopy and PI has several limitations: it is not possible to penetrate deeply into tissues, preventing imaging in plants with thicker roots than those of *A. thaliana*, which include most cultivated species and/or other model species.

Multiphoton microscopy is an alternative technology, not only because it allows deeper imaging but also because it is less harmful than confocal microscopy for in vivo tracking. Now used traditionally in the animal development biology field, for instance, for in vivo imaging of neural activity [6], the use of multiphoton microscopy in plant development biology is less common, despite its potential [7]. Thus, it is becoming urgent to develop a simple and powerful tool for the 3D imaging of thicker samples in plants. Recently, we obtained first images of root tips in rice using a multiphoton microscope to visualize the flow of root auxins [8].

In this work, we evaluated and identified key parameters to optimize and obtain reproducible 3D images of living root tips compatible with the use of fluorophores (CFP, GFP, mCherry, and YFP). We used this protocol to identify tissue-specific root markers during large-scale enhancer trap screening to demonstrate its usefulness and simplicity.

## Results

### A reproducible protocol for visualizing rice root meristem structure using PI staining and multiphoton microscopy

PI is traditionally used in *A. thaliana* to counter-color cell walls. In addition, PI is a vital dye that can be used to determine whether the observed cells are alive (cell wall staining) or dead (nuclear staining). We decided to use PI as a cell wall stain in our experiments. We noted great variability in our results during our first trials of visualizing the root tips after treatment with PI using a Multi-photon ZEISS LSM 7MP OPO [8]. We therefore sought to identify the critical parameters that influenced PI staining of cell walls.

**Fig. 1:**
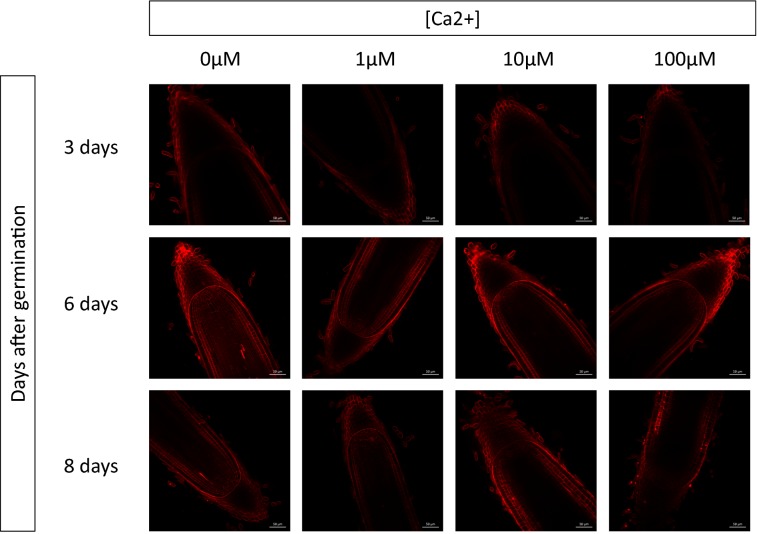
Effect of the Ca^2+^ concentration and the stage of development on the fluorescence of propidium iodide. Root tips of *Oryza sativa* cv Nipponbare rice seedlings (3, 6 and 8 days after germination) were incubated for 10 min in a 10 μM propidium iodide Merck H_2_O solution, with 0–100 μM CaCl_2_. The root tips were then rinsed twice in Merck H_2_O, and a median view (150 μm of the root tip surface) was always imaged with the same device settings (Wavelength: 1097 nm, Laser Power laser: 50, gain: 600, image resolution 1024 × 1024, speed 7, and average 8)

Calcium (Ca^2+^) is known to compete with PI for cell wall fixation [9]. We therefore used ultrapure water, tested the effect of different Ca^2+^ concentrations on PI cell wall fluorescence and hypothesized that the root developmental stage would also influence the cell wall composition and thus PI fixation. We then observed root tips 3, 6 and 8 days after germination, with Ca^2+^ concentrations ranging from 0 to 100 μM (Fig. 1). Briefly, root tips of *Oryza sativa* cv Nipponbare rice seedlings were incubated for 10 min in 10 μM PI. The root tips were then imaged with the same device settings (wavelength: 1097 nm, laser power laser: 50, gain: 600, image resolution 1024 × 1024, speed 7, and average 8).

Three days after germination, regardless of the concentration of Ca^2+^ used, the epidermis and the outermost root cap cells were the only cell layers visible. No internal layers, such as the endodermis or vascular tissues, could be recognized (Fig. 1). It was only 6 days after germination that root meristems with all cellular layers were distinguishable in the meristem, including the initials (Fig. 1). Eight days after germination, we could only separate from the outside of the root towards the inside, the epidermis, the sclerenchyma and the exodermis, as well as the three outermost cell layers of the root cap. At 6 days after germination, increasing the Ca^2+^ concentration led to a reduction of fluorescence and prevented the visualization of the walls of the most internal cellular layers beyond a concentration of 10 μM. The most important parameter appeared to be the root developmental stage, since 3 or 8 days after germination, it was almost impossible to detect cell wall fluorescence, regardless of the Ca^2+^ concentration. The best images were then obtained for plant root tips at 6 days old with no Ca^2+^ or a low (below 10 μM) Ca^2+^ concentration (Fig. 1).

**Fig. 2:**
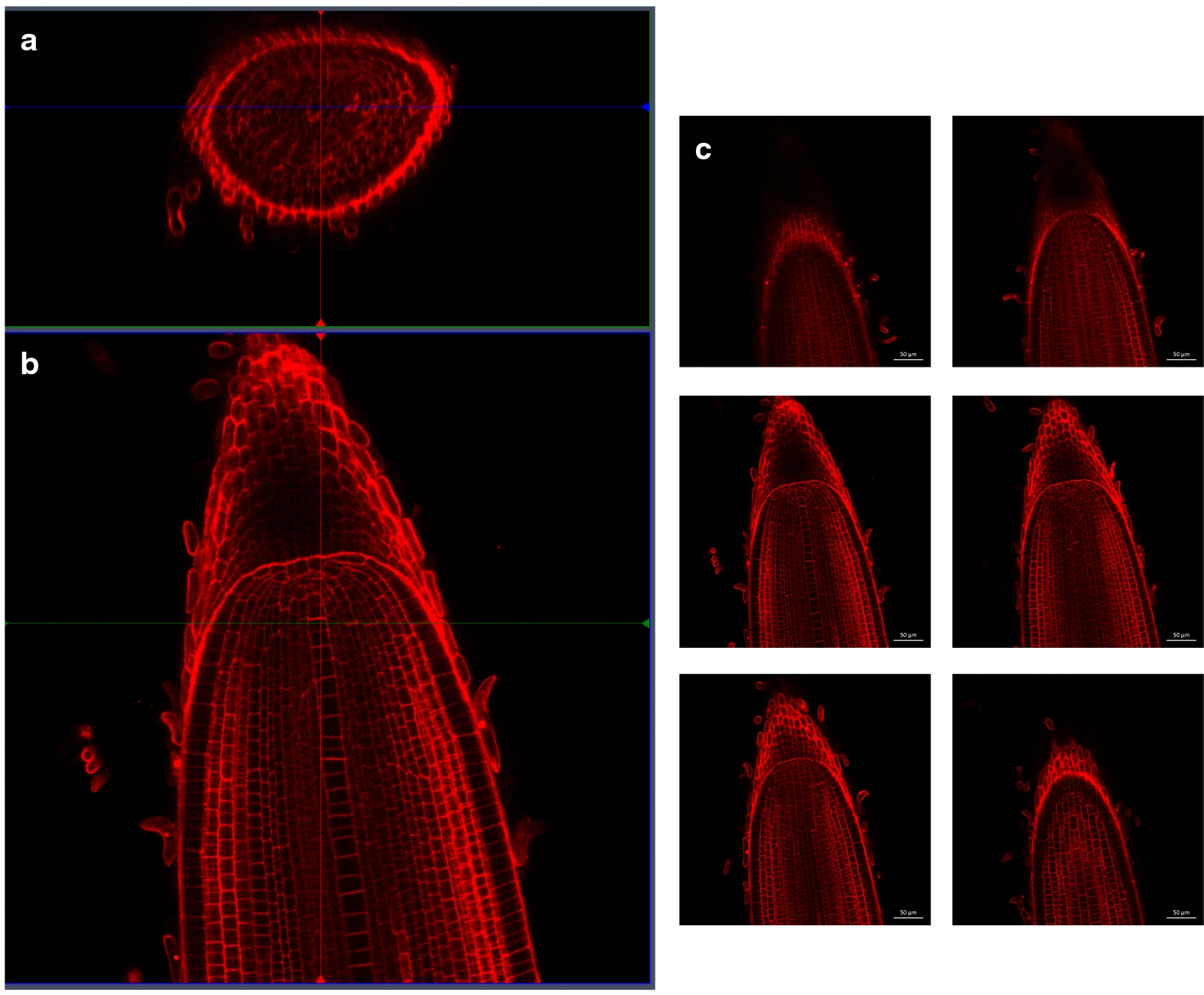
3D reconstruction of a 166 μm stack of an *Oryza sativa* cv Nipponbare rice root tip. The cell walls were counter-stained with 10 μM PI. **a** Radial view of the stack, after 3D reconstruction. **b** Longitudinal view of a median section of the root. **c** Successive longitudinal views of the root from the surface to the deepest view (respectively from **a**–**f**)

**Fig. 3:**
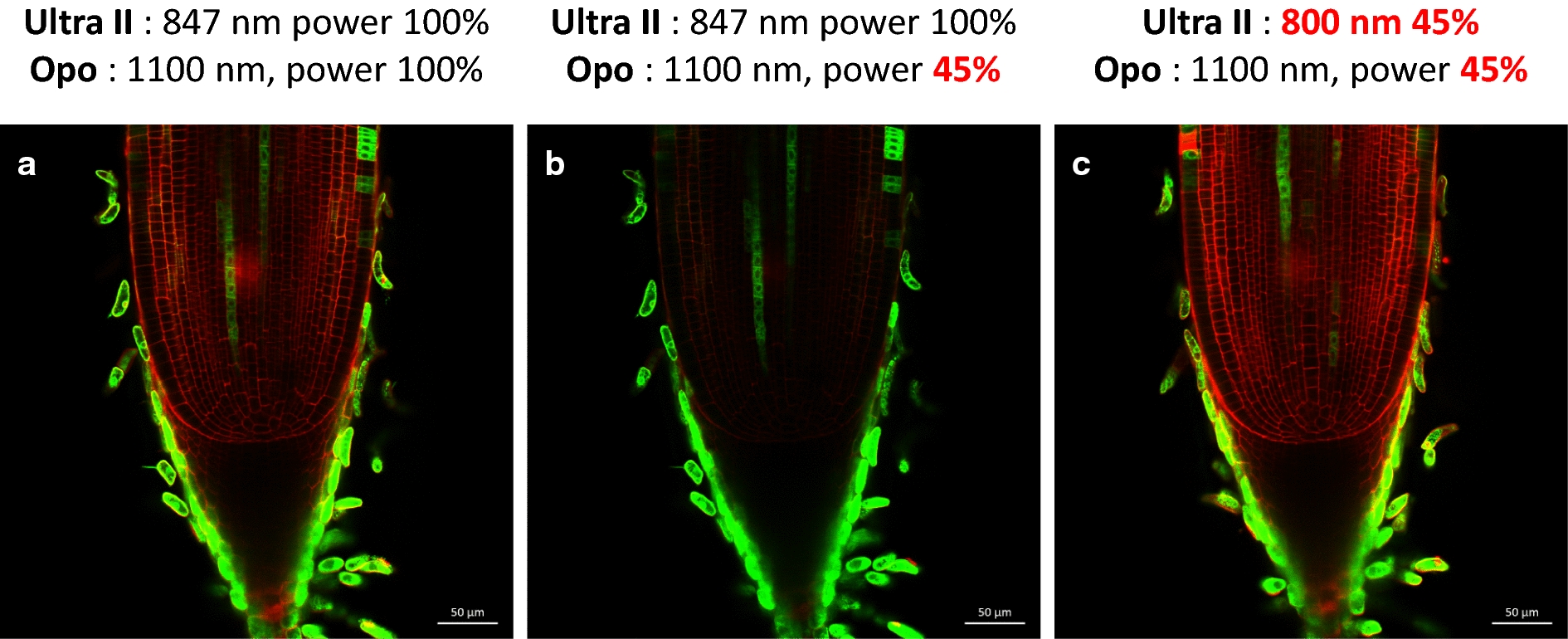
Combined effect of Ultra II and OPO laser wavelengths on the median views of the same enhancer trap line counterstained with PI (APR6). The cell walls are counter-stained with 10 μM IP. The same settings (gain: 600, image resolution 1024 × 1024, speed 7, and average 8) were used, except for the Ultra II and OPO laser wavelengths

Using these optimal parameters, we could easily obtain a complete 3D reconstruction of a rice root tip (Fig. 2 and Additional file 1: Video 1) at a depth of approximately 170 μm. A stack of images of a 6-day root tip colored with PI without Ca^2+^ was generated using a time step of 0.8 μm for a depth of 170 μm. The orthogonal cross-section view of the stack (Fig. 2a) allows the viewer, without any further processing, to distinguish all the root layers, including the most internal layer, the central metaxylem vessel (above the cross in Fig. 2a). In the median view (Fig. 2b), all cells, including initials that converge towards the root quiescent center, were clearly visible, and the stele/QC/root cap separation was also evident in this view. All central metaxylem proximal and distal cells could be recognized, indicating that the whole root meristem was visible (Fig. 2b). Furthermore, the root longitudinal view has a uniform fluorescence brightness (Fig. 3c), confirming the quality of the stack of images obtained.

In conclusion, the use of ultrapure water with a Ca^2+^ concentration lower than 10 μM on rice root tips 6 days after germination allows us to visualize in a reproducible way the cellular walls of a root tip in a median view at a depth of approximately 170 μm with the help of a multiphoton microscope.

### Simultaneous visualization of GFP-, CFP-, mCherry- and PI-stained cell walls inside living rice root meristems using a multiphoton microscope

In the second step, we sought to optimize the simultaneous visualization of GFP and PI inside the rice root meristem while minimizing the power of the laser used (Fig. 3). Indeed, by raising the laser power, it was always possible to improve the visualization of the fluorophores, but this approach also increased the risk of destroying root cells, leading to PI penetration and additional photosensitization. To jointly visualize IP and GFP fluorescence, we initially worked with two lasers, OPO (IP) and Ultra II (GFP), at wavelengths of 1100 nm and 847 nm, respectively. We therefore sought to minimize the energy transmitted to the root tips while optimizing the images obtained. An enhancer trap line (APR6 cf below) with a xylem-specific and root cap GFP expression profile was used to optimize the laser wavelength. With wavelengths of 1100 nm for the OPO (theoretical optimum for IP) and 847 nm for the Ultra II (theoretical optimum for GFP), it was possible to detect the cell walls and GFP of the APR6 line simultaneously, but only with both lasers powered at approximately 100% (Fig. 3a). The power of the OPO laser is dependent on the power of the Ultra II laser, and the energy of the Ultra II laser also depends on its wavelength. Decreasing the power of the OPO laser makes it impossible to visualize the cell walls (Fig. 3b). Using a wavelength of 800 nm, we obtained perfectly resolved images with lasers at 45% of their maximum power (Fig. 3c), although 800 nm was not the optimal theoretical wavelength for GFP. This effect was largely due to the fact that the maximum absolute power of the Ultra II laser was obtained at a wavelength of 800 nm. The fact that the power of the OPO laser is directly correlated with the power of the Ultra II laser allows the 800 nm wavelength to maximize the power level of the OPO laser. These parameters were therefore used for the joint visualization of GFP and PI in the following parts of the paper.

**Fig. 4:**
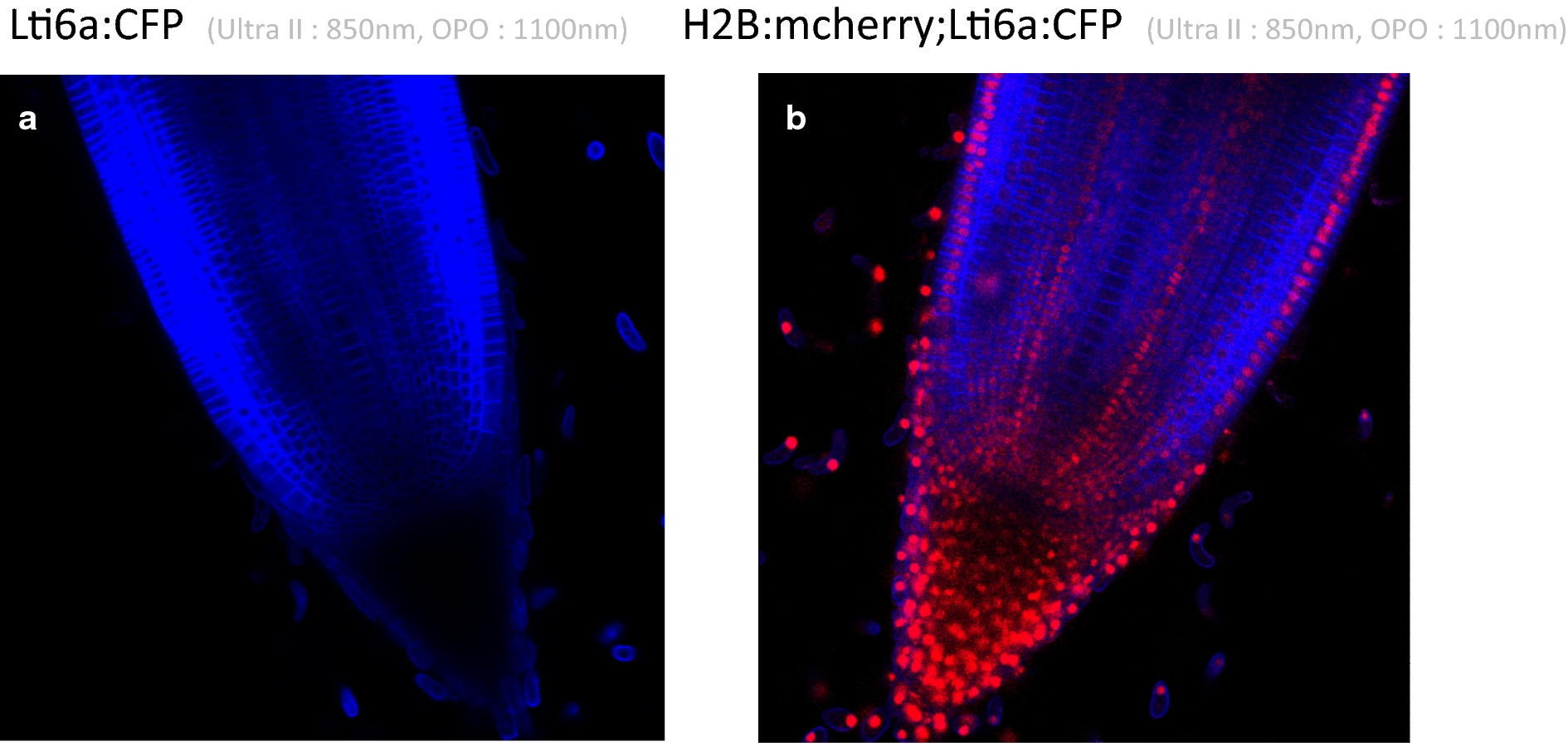
Root tip median views of transgenic lines expressing CFP (**a**) and CFP plus mCherry (**b**). **a** Median view of a pCSCMV:Lti6a:CFP line, Ultra II laser: 850 nm, OPO: 1100 nm. **b** Median view of a pCSCMV:Lti6a:CFP; pUBI:mCherry2x line, Ultra II laser: 850 nm, OPO: 1100 nm

In addition to GFP, other fluorophores are also used frequently, and we wanted to determine whether it was possible to visualize CFP and mCherry using the same protocol, while evaluating the optimal wavelengths for their detection. We therefore used transgenic rice lines transformed with the Lti6a:CFP construct and the Lti6a:CFP; H2B:mCherry constructs (Fig. 4). Lti6a is a membrane marker, and H2B:mCherry marks histones and therefore nuclei. CFP is detected using wavelengths of 850 nm for the Ultra II laser and 1100 nm for the OPO laser. All cell membranes were visible inside the root meristem (Fig. 4a), except inside the root cap. At this step, it is not clear if this effect is due to the Lti6a peptide itself or to the pCsCMV promoter driving Lti6a gene expression. It was nevertheless possible to jointly detect mCherry, which marks nuclei, and CFP using a wavelength of 850 nm for Ultra II and a wavelength of 1100 nm for OPO in median root meristem views. Again, CFP is not detected inside the root cap, but mCherry nuclei were visible inside the root meristem, including the root cap (Fig. 4b). Bright mCherry fluorescence was visible in all initials near the quiescent center but was only visible in the epidermis, endodermis and pericycle cell layers when leaving the active dividing part of the root meristem.

### Screening of enhancer trap lines and identification of tissue-specific GFP rice marker lines

**Fig. 5:**
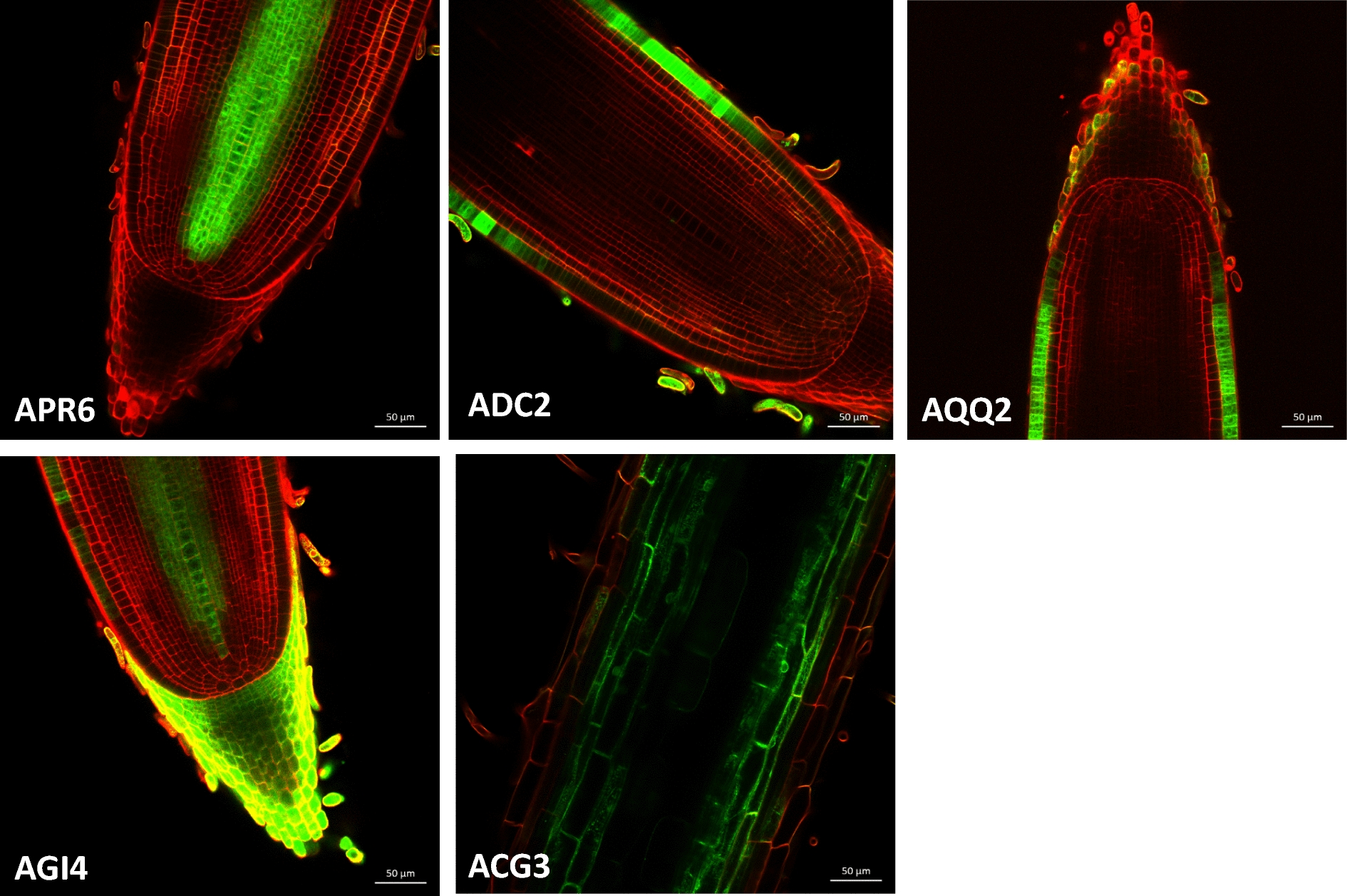
Median views of tissue-specific GFP OTL enhancer trap lines. The cell walls were counter-stained with 10 μM IP, Ultra II laser: 836 nm, OPO: 1100 nm. APR6 is a stele-specific expression line. AGI4 is a xylem- and root cap-specific expression line. AQQ2 and ADC2 are both epidermis-specific expression lines. ACG3 is a cortex-specific expression line in which the signal is visible only in the differentiated part of the root tip

The protocol is simple enough to be used routinely. To demonstrate its interest and simplicity, we used this protocol to partially screen an enhancer trap library from the Génoplante collection [10, 11]. Overall, 5500 progenies, with 20 seedlings per line, were sown in Petri plates with 20 ml of distilled water. After 10 days, the lines were observed under a binocular microscope with UV light and a long pass filter to visualize GFP. Of these 5500 progenies, 450 exhibited GFP expression in the root tips, and two GFP seedlings per line were transferred to the greenhouse and self-crossed. Of these 450 lines, 57 exhibited strong stable expression of GFP in the next generation and were sown in petri plates. The plates were all examined to precisely locate GFP within the root meristems. Finally, 5 of the lines had tissue-specific expression profiles (Fig. 5). APR6 had a stele-specific expression profile; ADC2 and AQQ2 exhibited epidermis-specific expression only in differentiated parts of the epidermis; AGI4 exhibited xylem- and root cap-specific expression, and ACG3 exhibited cortex-specific expression that was limited to differentiated tissues. This screening demonstrates the usefulness of the protocol for rapid root meristem imaging in rice.

## Discussion

PI binds to the pectin of plant cell walls [9] and does not penetrate the plasma membranes of living cells. PI is therefore both a vital stain and a cell boundary marker. The compatibility of PI with most fluorophores, such as GFP and CFP, allows it to be used as a nearly universal root counterstain, especially when in vivo meristem growth monitoring is required. Ca^2+^ ions compete with PI [9] for their fixation on cell walls, so this parameter must be controlled in the medium to improve PI staining/fluorescence and contrast. As Ca^2+^ is necessary for growth and is part of most conventional growth media, such as MS [12] and N6 [13], we recommend that a concentration of 10 μM should not be exceeded based on our results (Fig. 1). Such concentrations ensure that the fluorescence and visibility of PI are maximized, while remaining compatible with conventional growth media.

The effect of the developmental stage on the in-depth fluorescence of PI may be explained by differences in cell wall maturation and thus the presence of pectin, which is necessary for effective PI fixation [9]. Another non-exclusive explanation is the presence of casparian strips. Because PI does not penetrate living cells, it passes passively into the apoplast and is blocked by the mature casparian strips of the endodermis. Indeed, when the endodermis is mature in *A. thaliana*, PI can no longer penetrate beyond the distal cell wall [14, 15]. This property is also used in a test to determine whether a layer is a functional endodermis [14]. Rice also has casparian strips on the exodermis and endodermis [16]. This is an unlikely explanation for developmental stage effect on in-depth fluorescence of PI as rice endodermis and exodermis are probably not fully mature 6–8 days after germination. In practice, whatever the real reason for the effect of the root development stage on PI staining, our results suggest that the stage of development should be taken into account as a parameter for PI staining of new tissue cell boundaries. Increasing the PI concentration, that improve cell wall visualization, may also induced calcium deficiency. Using the concentration of 10 μM of PI is then a good compromise and should be compatible with root growth for short term experiments. A concentration of 10 μM of PI was used for instance in Arabidopsis cell ablation experiments [17].

The enhancer trap line library that was developed using the GAL4-UAS-GFP construct represents an important tool for functional analysis, and these libraries were first developed in *A. thaliana* [18]. These lines can be used as markers and specific tissue expression tools or to express any gene in a tissue-specific manner. Our laboratory generated such a collection of lines in rice and demonstrated that they can be used to trans-activate a reporter gene in a tissue-specific manner, as in *A. thaliana* [10]. A first partial screening of the collection had been initiated [10] but without the possibility of obtaining detailed GFP expression profiles using a confocal microscope [10]. In addition, we also noted a high silencing rate of GFP expression between the T1 and T2 generations. Thus, we decided to completely rescreen the GAL4-UAS:GFP T-DNA collection using seedlings of the T3 and T4 generations. Thanks to this large enhancer trap library and to the development of our protocol combining PI staining and multiphoton microscopy, we initiated root meristem imaging of 57 lines with stable GFP expression in the root tips. We obtained detailed GFP profiles of all of these lines at the tissue and cellular levels.

## Conclusions

It should now be possible to use PI and multiphoton microscopy in a large number of applications in plants with thicker roots. For example, it should now be possible to carry out experiments to monitor cell division [4] and cell ablation [17], such as in *A. thaliana*, to determine how internal root tissue is formed. It remains, difficult to determine how these tissues are formed based on anatomical data only and to infer the behavior of initials in these tissues, for instance in rice [16, 19, 20].

We successfully identified 5 lines with a tissue-specific expression profile. These lines are identity tissue markers that can be used in a complementary manner to other markers, such as anti-parietal antibodies [21], to determine ground tissue identity in rice [22]. These lines may also be used to obtain tissue-specific expression profiles via FACS [23]. These tissue-specific enhancer trap lines are now available to the rice community, together with root meristem imaging protocols. These protocols are now routinely used in the laboratory and optimized on a regular basis to facilitate root cell-scale functional analyses in this monocotyledon species.

## Methods

### Plant materials and plant growth conditions

Dehusked seeds of *O. sativa* cv Nipponbare were surface-sterilized in 70% ethanol for 1 min, then rinsed with sterile distilled water and disinfected by dipping in a 40% bleach solution diluted in distilled water with 0.4% Tween 80 (Sigma-Aldrich P4780-500 ml) for 30 min under gentle agitation at room temperature. The seeds were then rinsed three times with distilled water.

The seeds were sown on square Petri plates (Corning, 431,301; 20 cm × 20 cm) containing 250 ml of Murashige and Skoog (MS/2) solid medium (2.15 g/l of MS medium basal salt mixture (Duchefa Biochemie, M0221), 75 mg/l MS vitamin mixture (Duchefa Biochemie, M0409) and 8 g/l of type II agarose (Sigma-Aldrich, A6877). The Petri plates were then placed vertically in a culture chamber under controlled light (12-h photoperiod, light intensity 500 uEm-2s-1) and temperature (28/25 °C day/night) conditions for 6 days.

### Cloning and rice transformation

Two molecular constructs were generated via synthesis and cloned into the plasmid pUC57 (Genscript). Native recognition sites for the *Bam*HI, *Eco*RI, *Hind*III, and *Kpn*I restriction enzymes were systematically mutagenized in each construct to enable the stacking of both constructs into the binary plasmid pCAMBIA2300 (pCB2300). The first unit contains the maize ubiquitin-1 promoter (pUBI) that controls the expression of a fluorescent plasma membrane-localized fusion protein (ECFP-LTI6a) (Cutler et al., 2000) and the *Pisum sativum* pea 3A terminator (TER3A) (Fluhr et al., EMBO 1986). The second construct combines the CsCMV promoter (promCsCMV) driving the expression of a chimeric gene that contains a castor bean catalase intron (Ohta et al., 1990 Plan and Cell Physiology) and an Arabidopsis histone H2B (H2B, At5G22880) fused to mCherry and the nopaline synthase terminator (NOSter). The insert promUBI:ECFP-LTI6a-TER3A was released via double digestion with *Eco*RI and *Kpn*I, cloned into the binary vector pCAMBIA2300 (pCB2300), and linearized using *Eco*RI and *Kpn*I. The second construct was released via double digestion with *Bam*HI and *Hind*III and cloned into the linearized pCB2300-promUBI:ECFP-LTI6a-TER3A to generate a binary plasmid combining both constructs in one transferred DNA (T-DNA). The binary plasmids comprising the promUBI:ECFP-LTI6a-TER3A construct alone or in combination with the second construct promCsCMV:H2B-mCherry-NOSter were electroporated into *Agrobacterium tumefaciens* strain EHA105 or GV3101 for rice transformation, respectively. *Japonica* rice cv Nipponbare seed-embryo-derived callus was transformed according to the previously described protocol [24].

### Multiphoton microscopy settings

Root tips of a 1 cm length were cut and dipped in 10 μg/ml PI in H_2_O (Merck, VWR ref 1.15333.1000) for 10 min in the dark and then rinsed two times with H_2_O for 1 min. A microscopic chamber for stained root tips was assembled using a microscope cover glass and a microscope glass slide. For screening, we used an upright microscope (Multi-photon ZEISS LSM 7MP OPO) equipped with 2 lasers: chameleon Ultra II (680 and 1080 nm) and Chameleon Compact OPO (optical parametric oscillator) (1000 and 1300 nm). Z-stacks and images were acquired using a 20 × objective lens (W Plan Apochromat 1.0 NA Water, Zeiss). Each image was the average of eight lines. Images were acquired at 1100 nm for PI and 836 nm for GFP. The images were acquired and analyzed using the ZEN 2010 software (Zeiss).

### Enhancer trap T-DNA OTL collection screening for root meristem GFP expression

Ten seeds for a total of 5520 progenies from the OTL T-DNA insertion collection with the GAL4:UAS:GFP construction [10, 11] were sown into multi-well boxes (3 × 2). A total of 20 ml of sterile distilled water was added to each well, and the boxes were then transferred to a culture chamber under controlled light (12-h photoperiod, light intensity 500 uEm-2s-1) and temperature (28/25 °C day/night) conditions.

After 10 days, the Petri plates were observed under a Leica MZFLIII binocular magnifying glass under UV light using the GFP plus filter (LP 510 nm). One to two seedlings for each line with GFP fluorescence at the root tips were transferred to the greenhouse and self-fertilized. A total of 492 lines with GFP expression in the T3 generation were transferred to the greenhouse. Finally, 57 lines with strong GFP expression in the root tips were examined using multiphoton microscopy to locate GFP expression at the cellular level.

## Additional file

**Additional file 1: Video 1.** A stack of the full rice root tip. 166 μm of the stack were obtained by imaging successive views with a step of 0.8 μm, with a picture dimension of 1024 × 512 pixels. The cell walls are counter-stained with 10 μM IP, Ultra II laser: 836 nm, OPO: 1100 nm.
